# Surgical management of abdominal and retroperitoneal Castleman's disease

**DOI:** 10.1186/1477-7819-3-33

**Published:** 2005-06-07

**Authors:** Pascal Bucher, Gilles Chassot, Guillaume Zufferey, Frederic Ris, Olivier Huber, Philippe Morel

**Affiliations:** 1 Clinic of Visceral and Transplantation Surgery, Department of Surgery, Geneva University Hospital, Switzerland

## Abstract

**Background:**

Abdominal and retroperitoneal Castleman's disease could present either as a localized disease or as a systemic disease. Castleman's disease is a lymphoid hyperplasia related to human Herpes virus type 8, which could have an aggressive behavior, similar to that of malignant lymphoid neoplasm mainly with the systemic type, or a benign one in its localized form.

**Methods:**

The authors report two cases of localized Castleman's disease in the retroperitoneal space and review the current and recent progress in the knowledge of this atypical disease.

**Cases presentation:**

The two patients were young healthy women presenting with a hyper vascular peri-renal mass suggestive of malignant tumor. Both have been resected *in-toto*. One of them had an extensive resection with nephrectomy, while the second had a kidney preserving surgery. Pathological examination revealed localized Castleman's disease and surgical margins were free of disease. Postoperative course was uneventful, and after more than 5-years of follow-up no recurrences have been observed.

**Conclusion:**

Localized Castleman's disease should be considered when facing a solid hypervascular abdominal or retroperitoneal mass. A better knowledge of this disorder and its characteristic would help surgeon to avoid unnecessarily extensive resection for this benign disorder when dealing with abdominal or retroperitoneal tumors. Surgical resection is curative for the localized form, when complete, while splenectomy could be indicated for the systemic form.

## Background

*Castleman's disease *(CD) is a rare lymphoid disorder where pathogenesis is a lymphoid tissue hyperplasia related to chronic herpes virus infection. It has been described in nearly every lymph node basin since it first description by B. Castleman in 1956 [1,2]. Two basic pathologic types of this disease could be encountered: the hyaline vascular (HV) and the plasma cell (PC) types. The first tends to be localized in one lymph node and asymptomatic; the second has a more aggressive course and tends to be multifocal with systemic manifestations.

The authors' present two cases of localized Castleman's disease arising in the peri-renal space and review previous reports abdominal and retroperitoneal CD. A review of literature on Castleman's disease pathogenesis, clinical and radiological characteristics as well as its treatment is also included in this manuscript.

## Case presentation

### Case 1

A 33-years-old woman with no significant past medical history complained of abdominal right upper quadrant discomfort associated with an history of weight lost (8 kg over 2 months). Physical examination revealed a right upper quadrant mass on deep palpation. Routine hematology and blood biochemistry were normal. The patient was HIV1-2 negative. Chest and abdominal roentgenograms were considered normal. Abdominal ultrasonography (USG) revealed a large hypoechogenic mass, with regular border in the right anterior peri-renal space. Computed tomography (CT) scan showed a 10 × 8 cm mass with regular contour, containing small calcifications, which strongly enhanced with vascular contrast. The lesion was in contact with the right kidney and ureter. Surgery was planned with a preoperative diagnosis of malignant retroperitoneal tumor versus lymph node hyperplasia.

Through a right transverse incision, after mobilization of the duodeno-pancreatic bloc, a tumor was found in contact of the right kidney, ureter and caval vein. While the possibility of malignancy could not be neglected, the mass was dissected *en-bloc *with wide margin in peri-renal fat. To allow free surgical margin clinically a segment of the right ureter as well as inferior pole of kidney were also excised *en bloc*. A right nephrectomy was finally performed latter on as the ureteral defect could not be repaired.

Histopathological examination of resected specimen revealed localized Castleman's disease of the hyaline vascular type. Patient had smooth postoperative recovery and is free of disease more than 6 years after resection.

### Case 2

**Figure 1:**
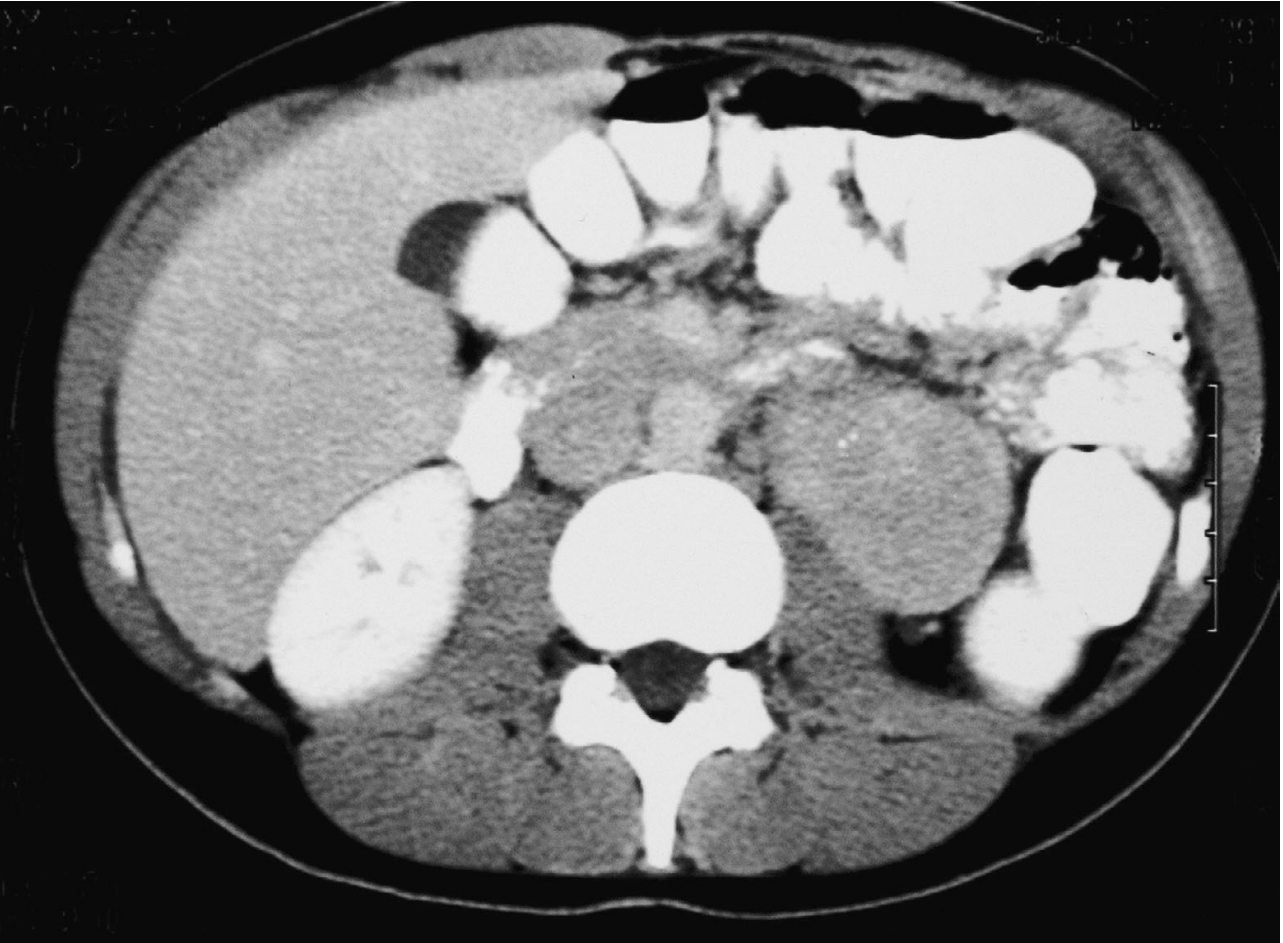
Computed tomography scanner showing a large left-pararenal mass (Hyalin-vascular type of Castleman's disease). Note the presence of microcalcification (white spot) within the mass.

**Figure 2:**
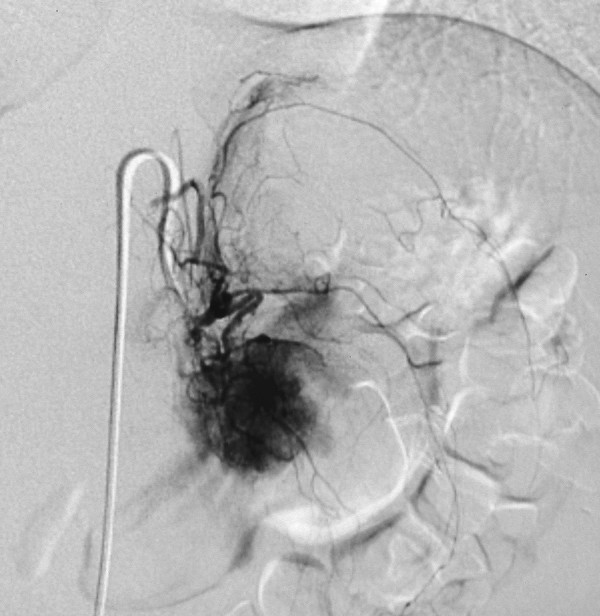
Arteriography showing the presence of a hypervascular mass (Hyalin-vascular type of Castleman's disease) with rapid flushing of the tumor. The feeding vessels originated from the aorta and left renal artery.

A 25-year-old woman with no significant past medical history, presented with post-prandial epigastric discomfort evolving over 2 years and post-prandial vomiting since 1 month. The patient reported 2.5 kg weight loss over 2 months. Physical examination revealed a left para-renal mass on deep palpation. Blood analyses were not relevant. CEA and CA19-9 were in the normal range. The patient was HIV1-2 negative. Chest and abdominal X-ray films were considered normal. An upper abdominal barium follow through was considered normal. Abdominal ultrasonography revealed a 6 cm diameter hypoechogenic mass in the left peri-renal space. CT scan showed a 6 × 7 cm mass, containing multiple small calcifications, which was highly hypervascular and regular in shape (figure 1). An arteriography confirmed the presence of hypervascularity with flushing of the mass (figure 2). Surgery was planned with a preoperative diagnosis of malignant retroperitoneal tumor.

At laparotomy a 7 cm diameter mass was found in the left anterior peri-renal space, just inferior to the renal artery. The lesion was completely excised with what seems, clinically, to be a capsule. No organ resection was needed.

Pathologic diagnosis was localized Castleman's disease of the hyaline vascular type. Patient had had a simple postoperative period, except of persisting lumbar pain, attributed to a small inferior renal infarct (confirmed by CT scan). The patient is free of disease 5-years after resection.

## Discussion

Castleman's disease (CD), also known as angiofollicular lymph node hyperplasia, was first reported by Symmers in 1921 [3]. This pathology was characterized by B. Castleman in 1956 as a benign lymph node hyperplasia resembling a thymoma [1,2]. Keller *et al *identified two pathologic types of CD in 1972 [4]. First the hyaline vascular (HV) type which present as a pathological and extensively hypertrophied lymph node. Macroscopically it appears as an encapsulated homogenous mass with an orange-yellowish color. Microscopically, it is characterized by giant lymph follicles centered on a central vessel with marked hyalinization. Follicles are surrounded by circumferentially arranged layer, in an onion skin feature, of small polyclonal B-lymphocytes. These pathologic lymph nodes present a strong hypertrophied vascular arborescence [4-8]. The plasma cell (PC) type has the same macroscopic aspect as HV type, but contains much more mature polyclonal plasma cells with a less marked hyalinization and vascularization [4,6-8]. It been shown that this two types histology are not always clearly separated and that mixed HV-PC types can also occur [7]. The histology of PC type is not specific of systemic CD and can be found in autoimmune disease, AIDS and in lymph nodes draining carcinoma, so it is imperious to exclude this condition before diagnosing CD of PC type [5,6]. This implies that serologic testing for HIV should be performed whenever a diagnosis of CD is contemplated[5,6].

The etiology of CD is related to chronic Human Herpes virus 8 (HSV8) [7] as HSV8 has been found in lymphoid cells in case of systemic form, or PC type, of Castleman's disease [9]. Its nature is not neoplastic as confirm by the fact that the lesion are made of a polyclonal proliferation [6,7]. It's seems that CD is the result of a chronic low grade inflammatory process triggered by latent infection with HSV8, which leads to lymphoid system hyperplasia [5-7]. Human herpes virus 8 (HSV8), also called Kaposi's sarcoma-associated herpes virus (KHSV) is the initiator of this chronic inflammation by establishing a chronic or latent infection in lymph nodes [7]. Chronic infection by HSV8 stimulates secretion of IL-6 which in turn induces a hyperplasic reaction of the lymphoid system [6,7]. While this lymphoid hyperplasia could be contained in one lymph node as in the localized form, which is mainly of HV type, it could also be generalized as in the systemic, or multifocal, form which is the predominant form for the PC type [6]. The patient's immunological status seems to play a major role in the development of these two forms. While localized form is encountered mainly in immunocompetent patients, the systemic form is found in patient with AIDS or other immunodeppression related either to immunosuppression or pathological state [6].

The localized form of CD arises predominantly in the mediastinum, where it was first described by B. Castleman [1]. However, it can be found in the neck, abdomen, axilla, inguinal region and in virtually all lymph node area [5,7,8,10,11]. Even non-nodal tissue could be involved, as is has been described in: lung, pancreas, breast, adrenal gland, muscle and other extremely rare locations [4,7,10,11]. Testa *et al *[11] have reported the location of 315 cases of localized CD, 65% were in the mediastinum, 16% in the neck, 12% in the abdomen, 3% in the axilla and 4% in diverse locations.

A literature review of the abdominal and retroperitoneal case of localized HV type of CD has been done. In 1992, 54 abdominal and retroperitoneal cases were reviewed by Seco *et al *[5]. Now 195 cases of localized CD have been reported, in the world literature, arising in the abdomen and retroperitoneum. Of these 195 cases, 122 (63%) were in the retroperitoneum and 73 (37%) in the abdominal cavity (Table 1). Of the 122 lesions localized in the retroperitoneum, 24 (20%) were in the peri-renal region, as were our cases. Nearly all this lesions were derived from lymph node tissue, but 5 of these 195 cases (2%) seem to have originating in extra lymphoid organ. Three pancreatic, and one each of splenic and adrenal CD have been described [58-60,65,80].

**Table 1:** Abdominal and retroperitoneal case of localized Castleman's disease (HV type)

***Location***	***Number of cases***	***Authors***
**Retroperitoneu**m	97	Seco *et al *[5], Bapat *et al *[12], Yamakita *et al *[13], Morishita *et al *[14], Genoni *et al *[15], Ng *et al *[16], Baikovas *et al *[17], Johnson *et al *[18], Martino *et al *[19], Guglielmi *et al *[20], Ziv *et al *[21], Gheysens *et al *[9], Halvic *et al *[22], Herrada *et al *[23], Furuhata *et al *[24], Ebine *et al *[25], Sadamoto *et al *[26], Gravalos *et al*. [27], Iwamoto *et al *[28], Sanna *et al *[29], Singletary *et al *[30], Curciacrello *et al *[31], Gonzalez Sanchez *et al *[32], Schutz *et al *[33], Perez Garcia *et al *[34], Irsutti *et al *[35], Parez *et al *[36], Buchanan *et al *[37]
*Peri-renal*	24	Ebisno *et al*. [38], Inoue *et al*. [39], Takihara *et al*. [40],,Feudis *et al *[41], Barret *et al*. [42], Okada *et al *[43], Present two cases
Mesentery	27	Seco *et al*. [5], Barki *et al*. [44], Hung *et al*. [45], Schroff *et al*. [46], Makipernaa *et al*. [47], De Heer-Groen *et al *[48], Parez *et al *[36], Neerhout *et al *[49], Powel *et al *[50], Burke *et al *[51]
Greater omentum	3	Volta *et al *[52], Kiguchi *et al *[53]
Gastric	2	Kiguchi *et al *[53], Yebra *et al *[54]
Peri-pancreatic	5	Kiguchi *et al *[53], Rotman *et al *[55], Brossard *et al *[56], Inoue *et al *[39], Erkan *et al *[57]
Pancreatic	3	Chaulin *et al *[58], Corbisier *et al *[59], Lepke *et al *[60]
Porta hepatitis	5	Rahmouni *et al *[61], Farkas *et al *[62], Peck *et al *[63], Cirillo *et al *[64]
Adrenal gland	1	Debatin *et al *[65]
Pelvis	27	Seco *et al *[5], Latte *et al *[66], Daley *et al *[67], Boxer *et al*. [68], Tsukamoto *et al *[69], Luburich *et al *[70], Isik *et al *[71], Ylinen *et al *[72], Schwartz *et al *[73], Mondal *et al *[74], Kiguchi *et al *[53], Calvo Villas *et al *[75], Mac Donald *et al *[76], Fields *et al *[77], Kkasantikul *et al *[78], Halvic *et al *[22], Murphy *et al *[79]
Spleen	1	Taura *et al *[80]

***Total of cases***	***195***	

The clinical presentations of CD differ greatly between the localized and the systemic forms (Table 2). The first appears in young generally healthy patients and cause few symptoms [6,7]. Abdominal and retroperitoneal locations, as were our cases, can be associated with mass effect symptoms related to compression of adjacent organs. This could present as: post-prandial discomfort, anorexia, vomiting, weight loss, urinary retention and abdominal or lumbar pain [80]. Systemic, or multifocal, CD is associated with systemic disturbance as anemia, increased erythrocyte sedimentation rate, polyclonal hypergammaglobulinemia, hypoalbuminemia and thrombocytopenia which all can be associated with a specific symptoms [6-8]. The clinical picture includes asthenia, fever, weight loss, generalized lymphadenopathy, hepatomegaly, splenomegaly, peripheric edema, pleural effusion, impaired renal function and sometimes polyneuropathy [6-8]. Rarely POEMS syndrome (Polyneuropathy, Organomegaly, Endocrinopathy, M protein and Skin change) or amyloidosis may be associated to systemic CD [6-8].

**Table 2:** Clinical forms of Castleman's disease

	Localized form	Multifocal form
Mean age (years)	3^rd ^decade	6^th ^decade
Clinical signs	Incidental mass effect	Systemic symptoms
Localization	Mediastinum, cervical or abdominal, etc...	Multifocal, mostly peripheric lymph nodes
Histologic type	HV, rarely HV-PC	PC
Treatment	Surgical resection	Corticosteroids, chimiotherapy, radiotherapy
Prognosis	Excellent: 100% survival at 5 years	Poor: median survival of 30 months
Recurrence after treatment	Extremely rare, related to incomplete resection	Nearly always
Association	Rarely lymphoma	Frequent: AIDS, Kaposi's sarcoma, lymphoma and myeloma

Table adapted according to references: [5, 6, 7, 11]

Radiographic characteristics of CD are non specific, but some features could help to suspect the diagnosis (Table 3) [77,80]. Plain radiographic finding includes a mass effect and in nearly 30% of localized form calcifications harboring a radial arrangement or star-shaped calcifications which is said to be characteristic of CD [77]. Ultrasonography (US) usually demonstrates a hypoechogenic and homogenous mass with quite clear delimitation [8,30,77]. US can show central areas of sharp acoustic shadowing due to calcification [30]. CT scan show a solid, homogenous and well delimited mass which enhance with vascular contrast as a results of hypervascularity [5,30,77,80]. It can also show star-shaped microcalcifications which are quite specific on pre-contrast images [5,27,77]. Post-contrast IV study, while demonstrating dense enhancement of the mass, could demonstrate a central stellate scar [30,80]. Angiography shows a strongly hypervascular lesion, which present a dense and homogenous flush during the capillary phase [5,6,8,40,53,77,80]. This flush begins in periphery to become diffuse whiting the mass and is specific for the HV type of CD [77,80]. It can also demonstrate hypertrophied feeding vessel, an useful information when resection is plan [8,53,77,80]. Magnetic resonance imaging (MRI) characteristics of CD are: hypodense mass on the T1 weighted study and hyperdense lesion on the T2 weighted image sometimes with star-shaped calcifications [60,77,80]. Gadolinium injection produces an enhancement which appear in periphery to become diffuse similarly to the flush observed during angiography [77,80]. All these radiological finding are not specific but some like the star-shaped calcifications and the type of hypervascularisation are quite specific and should alert clinician to the possibility of CD. In summary, in front of an abdominal or retroperitoneal mass which is well delimited, homogenous and harbor star-shaped calcifications and hypervascularisation associated with typical flush, the diagnosis of CD should be strongly suspected [53,77,80]. The differential radiological diagnosis is mainly malignant neoplasm because of the hypervascularity [40,77], and the fact that 80% of the retroperitoneal tumor are malignant [40,81,82]. The major tumors found in the retroperitoneum are soft tissue sarcoma (liposarcoma, fibrosarcoma, leiomyosarcoma, neurofibrosarcoma, undifferentiated and rabdomyosarcoma) which are frequently heterogeneous mass and show necrosis on CT scanner; vascular tumor (hemangiosarcoma and lymphangiosarcoma) which are cystic and of liquid density on USG and CT scanner; and the lymphoma which generally presents as multiple adenopathy and homogeneous mass on CT scanner [30,83-86]. Urological tumors like seminoma, prostatic cancer and teratoma tends to give rise to metastatic disease in the retroperitoneum in the form of adenopathy which are generally multiple [86]. While a preoperative diagnosis of CD is difficult to obtain, fine needle biopsy is not a definitive tools because of there low specificity and the differential diagnosis with lymphoma is impossible by this approach [77,88]. In addition to be non-specific and rarely yielding enough useful tissue, needle biopsy is associated with tumoral seeding with reported frequency of 1/40 000 to 1/1 000 biopsy [83,89]. Thus when surgery is planned, indication for fine needle biopsy should be carefully deliberated, while an open biopsy could always be done during surgery [83].

**Table 3:** Radiological characteristics of Castleman's disease

	**Non specific signs**	**Specific signs**
Radiography	calcification.	Star-shaped calcification.
Echography	Hypoechogenic and homogenous mass. Central areas of acoustic shadowing (calcification).	
CT scanner	Tissue density, Homogenous and well delimited mass. Contrast enhancement beginning in periphery.	Star-shaped microcalcifications Star-shaped scar on post-contrast study.
Arteriography	Hypervascularity with hypertrophied feeding vessel.	Flush beginning in periphery to become diffuse and homogenous during capillary phase.
MRI	Hypodense on T1 and hyperdense on T2. Contrast enhancement beginning in periphery.	Star-shaped microcalcification or star-shaped hypodense signal on T2.

Table adapted according to references: [5,9,27,30,34,42,48,65,]

Treatment of CD differs between localized and multifocal, or systemic, forms. The standard therapy of localized form is surgical excision which is curative when resection is complete and *en-bloc*. No recurrences have been reported after total excision in the literature as it was the case with our cases [5-7,11,38,40,53,82,90]. Because these lesions are highly vascularised, embolization before surgery could be helpful to minimize blood lost during surgery [5,53]. The problem that is faced during resection is to resolve the differential diagnose between a malignant pathology and CD. Macroscopically it is nearly impossible because CD lesions harbor dense fibrous adherences to adjacent organ and hypervascularization typically seen in malignant pathology [5,38,82,90]. For this reason peroperative diagnosis by open biopsy is helpful, and necessary in case of CD suspicion. It enables one to avoid extensive resection and especially resection of nearby organ for this benign disorder which does not invade adjacent organ even in case of tight contact [38,82,83,90]. The five years survival after resection is nearly 100% for the localized form [6]. Recurrences have rarely been reported generally when excision was incomplete [6,38,82,90]. For the systemic form no curative therapies have been found yet. Corticotherapy, immunosuppressive drugs, chemotherapy and radiotherapy have been tried without any convincing results [5-7,91,92]. The prognosis of this form is poor with a median survival of 30 months [6]. However recently, the reports of improvement and prolonged survival after splenectomy have been reported with steroid therapy and chemotherapy this could change the prognosis of the systemic form in the future [91,92]. Of importance from the surgical point of view is that in these cases the remission was obtained after splenectomy only [91]. The systemic, PC type, and extremely rarely the localized, HV type, forms are associated with malignant disorder such as Kaposi's sarcoma, malignant lymphoma and myeloma. This association is stronger in HIV positive patients with CD, for example Kaposi's sarcoma is associated to 13% of case of PC type CD in HIV negative and in 75% of case when HIV positive patients. The association with Kaposi's sarcoma could be explained by the fact that HSV8 is cause in the pathogenesis of these two disorders [7]. These neoplasms can appear as late as 8 years after the diagnosis of CD, so a long term follow-up is required in patient in whom the diagnosis of CD has been established [6].

## Conclusion

Castleman's disease (CD) is a rare lymphoid disorder, where etiology is related to Human herpes virus 8. CD could present in two forms: the localized (hyaline vascular type) and the systemic (plasma cell type). The localized hyaline vascular form has a unique indolent lymph node hyperplasia; which can be found in the abdomen, retroperitoneum or any lymph node basin; as a solitary mass. Localized CD is radiologically nearly undistinguishable from malignant neoplasms but some characteristics are quite specific, like the type of hypervascularization and the star-shape microcalcifications. A good preoperative work-up and an open biopsy during surgery, for abdominal and retroperitoneal mass if no diagnosis has been establish, can help to avoid extensive resection when facing this benign disorder. Complete surgical excision is curative; recurrences have only been described after incomplete resection. The prognosis is excellent with a five years survival of nearly 100%.

## List Of Abbreviations

CD = Castleman's Disease

HV = Hyaline vascular type (of Castleman's disease)

PC = Plasma cell type (of Castleman's Disease)

CT scan = Computed tomography scanner

US = Ultrasonography

## Competing Interests

No competing interests have to be reported for this work and manuscript by any of the authors or institution.

## Authors' Contributions

**PB**: Study design, literature review, patient's follow-up, medical charts review, and manuscript.

**GC**: Literature research and review.

**GZ**: Medical charts review.

**FR**: Literature review and manuscript.

**OH**: Patient's surgical management, manuscript review.

**PhM**: Manuscript review.
